# Synthesis, Characterization and Optimization of Hydrothermally Fabricated Binary Palladium Alloys PdNi_x_ for Use as Counter Electrode Catalysts in Dye Sensitized Solar Cells

**DOI:** 10.3390/ma12193116

**Published:** 2019-09-25

**Authors:** Nyengerai Zingwe, Edson Meyer, Johannes Mbese

**Affiliations:** 1 Fort Hare Institute of Technology, University of Fort Hare, Alice 5700, South Africa; emeyer@ufh.ac.za; 2 Department of Chemistry, University of Fort Hare, Alice 5700, South Africa; jmbese@ufh.ac.za

**Keywords:** hydrothermal, counter electrode, palladium, alloy, dye sensitized solar cell

## Abstract

The hydrothermal synthesis, characterization and optimization of binary palladium alloys PdNi_x_ is hereby presented in this work. Comparison of the reduction capability of the developed PdNi_x_ alloys intended for use as alternative counter electrode catalysts in dye sensitized solar cells was made relative to the standard platinum counter electrode catalyst as well as the carbon supported PdNi-rGO sample. Optimization was accomplished through varying the molar ratio of the reagents. The unsupported PdNi_3_ sample produced the highest catalytic efficiency with reduction current density, peak to peak potential difference and charge transfer resistance of 35 mA cm^−2^, 0.15 mV and 0.47 Ω respectively. Obtained results show that the unsupported PdNi_3_ alloy was catalytically more effective than the platinum and PdNi-rGO thus could be a viable replacement in dye sensitized solar cell counter electrodes.

## 1. Introduction

Energy sufficiency is amongst the critical and essential requirements for the economic development of any nation [1]. African nations with poor grid infrastructure cannot meet the electricity capacity regularly required by customers. To that end, the use of solar energy should be aggressively adopted so that consumers do not heavily rely on non-renewable sources. Solar energy is one such renewable energy source which could readily supply the required energy to alleviate grid pressure. However, electricity generation from solar is a very expensive, and low efficiency process [2]. In order to improve the process of electricity generation from solar, newer technologies must be invented or significant improvement of the existing technologies is required. Presently the silicon based solar cell is the highest performing and stable technology utilized for electricity generation. Despite significant research into improving the functionality of the silicon solar cell, its efficiency is modest at 22.3% [3] whilst also possessing a significantly complex process of silicon purification which is expensive. Nevertheless, the silicon cell is the most effective and reliable photovoltaic technology available currently. The dye sensitized solar cell is amongst the other photovoltaic technologies which has considerable advantages including ease of operation, low fabrication cost, modest efficiency of 13.8% [4] and environmental compatibility. The dye sensitized solar cell consists of a mesoporous semiconductor photoanode, dye sensitizer, redox electrolyte and the counter electrode [5]. The counter electrode plays a crucial role of reducing the oxidized electrolyte ion thus facilitating electron transportation from the outer circuit to the dye so as to complete the solar to electricity generation cycle. Platinum has always been the most sought after counter electrode for dye sensitized solar cells because of its high electrocatalytic capability [5]. Gratzel utilized a platinum counter electrode in the first assembled DSSC yielding power conversion efficiency between 7.1–7.9% in simulated solar light and 12% in diffuse daylight [6]. However, platinum is afflicted by several issues including being expensive, high charge transfer resistance stemming from average conductivity as well as being unstable in the corrosive iodine environment. These factors play significant roles in limiting its capability over time whilst also making the DSSC a cost ineffective technology.

One appreciable solution to the challenges associated with the platinum counter electrode is the development and use of metallic alloys. Charge transfer between the alloyed components creates a synergistic effect which facilitates a greater number of catalytically active sites for interaction with the electrolyte [7]. This effect thus enhances the electrocatalytic ability of the metallic alloys over the platinum counter electrode. Oh et al. [8] fabricated a CoPd alloy which exhibited 5.47% PCE compared to 5.17% for the platinum counter electrode based DSSC. More significantly the CoPd alloy CE had charge transfer resistance and reduction current density of 1.17 Ω and 1.341 mA respectively signifying excellent electrocatalytic activity and conductivity. Other alloys which have outperformed the Pt counter electrode include the NiCoS4 alloy CE developed by Huo et al. [9] which had reduction current density and charge transfer resistance of 0.774 Ma cm^−2^ and 2.2 Ω respectively compared to 1.002 and 5.2 Ω for the platinum counter electrode. Power conversion efficiency for the sulphide alloy was 8.8% compared to 8.1 for platinum. Anuratha et al. [10] reported on the NiCo2S4 nanocomposite which produced lower charge transfer resistance and higher power conversion efficiency at 0.85 Ω cm^2^ and 7.36% respectively compared to 1.27 Ω cm^2^ and 7.23% PCE for the platinum counter electrode. Wang et al. [11] also developed a PtNi alloy counter electrode, which exhibited significantly better electron transfer compared to the pristine platinum counter electrode. Resultantly the charge transfer resistance for this PtNi alloy was 0.744 Ω cm^2^ compared to 18.320 Ω cm^2^ for platinum. The high charge transfer resistance for the platinum electrode makes the dye sensitized solar cell become more susceptible to higher electron hole recombination rates. Thus, in order to minimize the electron-hole recombination’s as well as not sacrifice high efficiency for cost effectiveness we intent to develop and optimize binary palladium alloys PdNix which possess high catalytic activity ensuring greater functionality of the DSSC. Since palladium has similar physical and chemical properties to platinum thus it would be tasked with facilitating greater electrocatalytic activity whilst the nickel will shore up the electron density on the palladium atom through charge transfer. Fabrication methods for metallic alloys include dry plasma reduction [12], galvanostatic displacement [13], cyclic voltammetry method [14], and hydrothermal synthesis [15]. The hydrothermal method produces good quality crystals which are required for producing highly effective counter electrodes. In that regard this work intents to develop and optimize PdNi_x_ alloys for use as counter electrodes in dye sensitized solar cells. Furthermore, in order to eliminate the several problems associated with the iodine electrolyte which include being very corrosive and volatile a ferrocene electrolyte was utilized during the electrochemical analysis. Since the function of the counter electrode is to facilitate the reduction of the electrolyte, inference into the capabilities of the developed counter electrode samples was derived from the electrochemical metrics such as reduction current density, peak to peak potential difference and charge transfer resistance, which give insight into the catalytic performance of the samples.

## 2. Materials and Methods 

**Figure 1:**
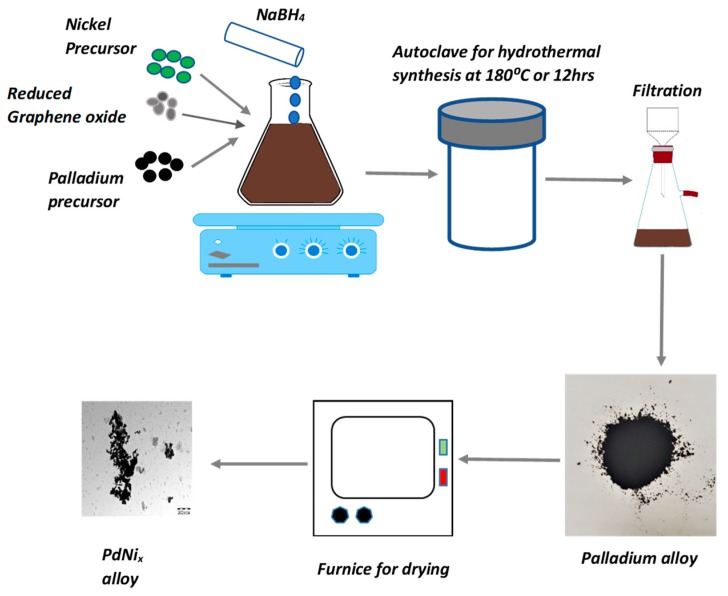
Procedure for hydrothermal synthesis of binary palladium alloys PdNi_x_.

The fabrication procedure for the binary palladium alloys PdNi_x_ included the initial preparation of the precursor solutions which were obtained by dissolving appropriate amounts of nickel nitrate hexahydrate Ni(NO_3_)_2_∙6H_2_O and potassium tetrachloropalladate (K_2_PdCl_4_) in 50 mL deionized water. The exact amounts of the reactants mixed together in order to develop the binary alloys are listed in Table 1. The produced mixture then underwent vigorous ultrasonication for 1 h so as to attain homogeneity. Under intensive stirring 5 mL of 2 mM sodium borohydride was added to the precursor solution resulting in a black mixture. The as-synthesized mixture was then transferred into a 100 mL stainless autoclave for hydrothermal synthesis. The hydrothermal synthesis was undertaken at 180 °C for 12 h which resulted in a black palladium alloy precipitate. The as synthesized black palladium precipitates underwent filtration followed by thorough washing with deionized water and ethanol. Subsequent drying of the synthesized alloy at 100 °C for 2 h was conducted in a furnace prior to characterization. Figure 1 shows the sequential procedure utilized for the hydrothermal fabrication of the palladium alloys. Fabrication of the carbon supported PdNi-rGO sample followed the same procedure as that utilized in the synthesis of unsupported PdNi_x_ samples except for the addition of reduced graphene oxide. The amount of the reagent’s utilized for the fabrication of the PdNi_x_ alloys were derived from previous works such as Zhang et al. in which maximum catalytic activity by palladium-gold alloys was observed when the palladium quantity was low with the best ratios between Pd:Au in the range from 0.1–0.3 [16].

Evaluation of the morphological, structural and electrochemical properties of the developed alloys was conducted using various techniques. Phase composition of the prepared PdNi_x_ alloys was conducted using XRD spectre obtained from a Bruker D8 Advanced X-ray Diffractomer (XRD) (Bruker, Madison, WI, USA) with a Cu anode, generating ration of wavelength 1.544 Â and operating at 40 kV and 40 mA. XRD spectre were obtained in the 2θ range from 15 to 90°. Field emission scanning electron microscope (FE-SEM) Zeiss Auriga SEM (Carl Zeiss, Oberkochen, Germany) equipped with EDS Smart SEM software was utilized for conducting morphological analysis of the palladium alloys as well as their elemental composition. Electrochemical analysis was conducted using a Bio-Logic VMP-300 potentiostat (Bio-Logic Science Instruments, Knoxville, TN, USA) controlled by the EC lab V10.37 software. Cyclic voltammetry curves were obtained in the 0–0.4 V potential range at scanning rates ranging from 0–100 mV s^−1^. Electrochemical impedance spectroscopy curves were obtained in the frequency range from 0.01–100 kHz at 0 V bias with an amplitude of 10 mV. The electrolyte utilized for the electrochemical analysis consisted of 0.1 M ferrocene, 0.06 M ferrocenium hexafluorophosphate and 0.5 M tert-butyl pyridine in acetonitrile.

## 3. Results and Discussion

**Figure 2:**
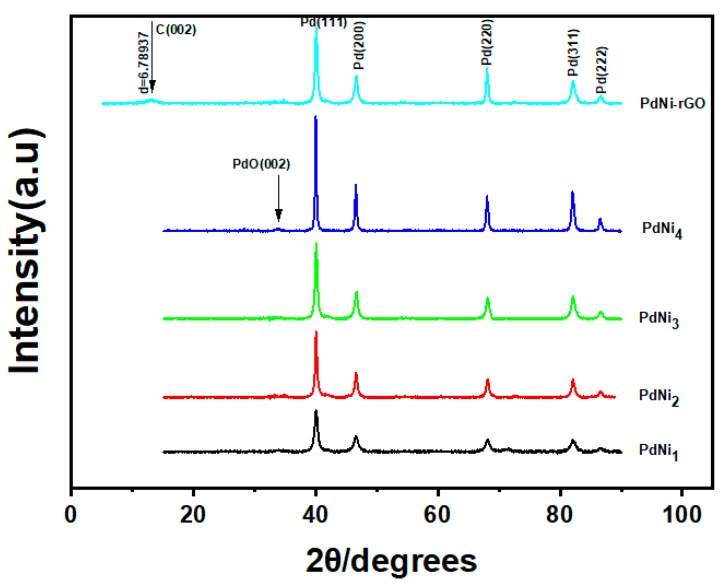
Scanning electron microscopy (SEM) images for the synthesized binary palladium PdNi_x_ alloys.

Figure 2 shows the XRD spectra for the as -synthesized PdNi alloys. The diffraction patterns for all the PdNi alloys had peaks at 2θ values of 40.1, 46.6, 68.1, 82.1 and 86.6 corresponding to the (111), (200), (220), (311) and (222) lattice planes of face centered cubic palladium. PdNi_4_ also exhibited a peak at 33.5° which was attributed to the (002), plane of PdO. Average particle size for the for the as-synthesized PdNi alloys estimated using the Scherer equation at 2θ value of 40.1° were 16.89, 20.6, 19.81, 19.12 and 28.92 nm for PdNi_1_, PdNi_2_, PdNi_3_, PdNi_4_ and PdNi_5_ respectively. Since no peaks were observed for nickel or nickel oxide thus formation of the binary palladium alloys can be deemed as having been successful. High intensity peaks for the Pd (111) lattice plane shows that the crystal growth was oriented in that direction. The peak observed at 14.4° for PdNi-rGO was assigned to the (002) plane of carbon from the reduced graphene oxide.

**Figure 3:**
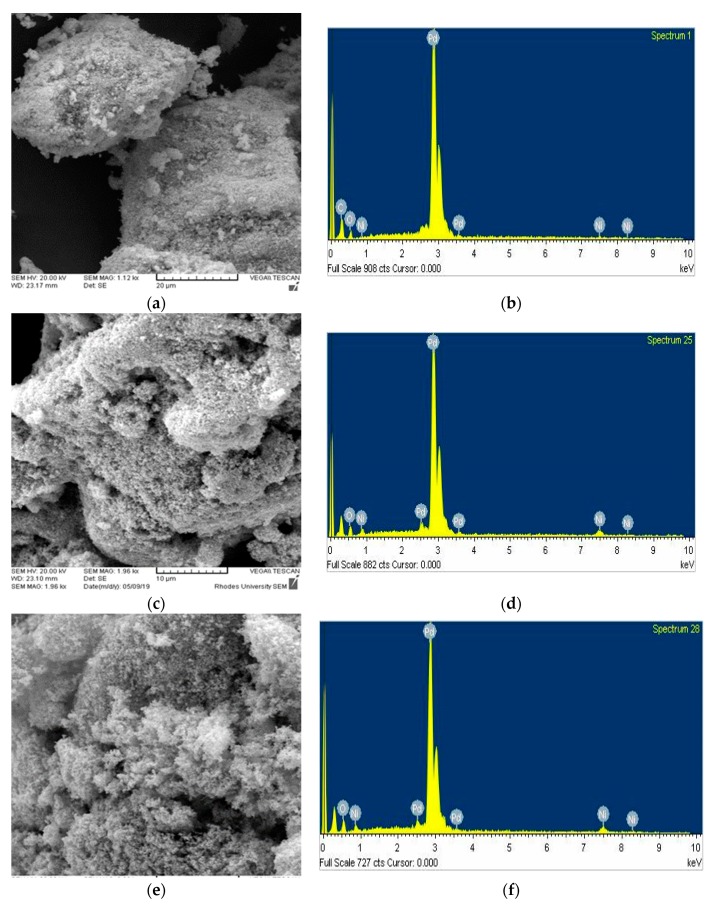

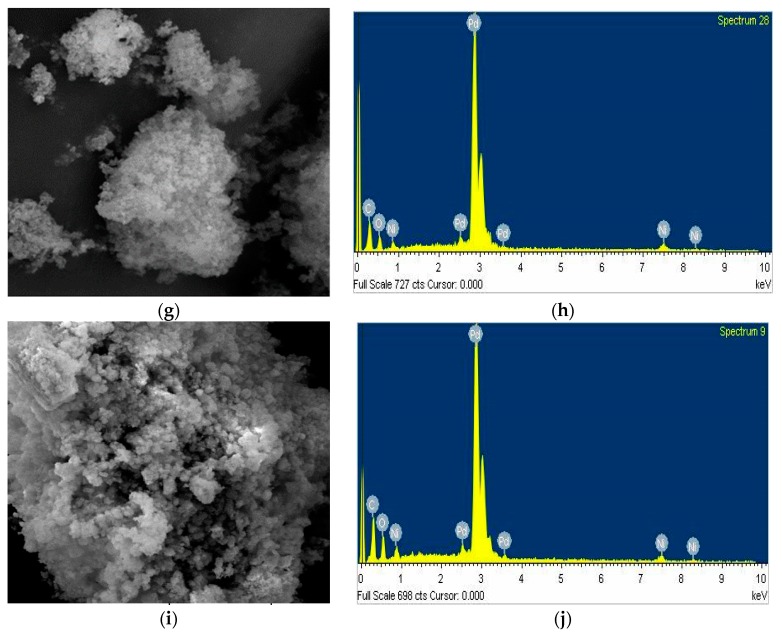
Scanning electron microscopy (SEM) images for the synthesized binary palladium PdNi_x_ alloys (**a**) PdNi_1_, (**c**) PdNi_2_, (**e**) PdNi_3_, (**g**) PdNi_4_, (**i**) PdNi-rGO, Energy dispersive spectroscopy (EDS) images for (**b**) PdNi_1_, (**d**) PdNi_2_ (**f**) PdNi_3_, (**h**) PdNi_4_, (**j**) PdNi-rGO.

The morphology of the developed PdNi_x_ alloys was analyzed using scanning electron microscopy (SEM). Figure 3 shows the SEM images of the binary palladium-nickel alloys as well as the elemental composition of the samples from energy dispersive spectroscopy (EDS). The EDS results show that all the alloys primarily consisted of palladium, and nickel as well as carbon for the PdNi-rGO sample. Particles of all the palladium alloys exhibited a spherical structure with agglomeration being observed for all the as-synthesized palladium alloys which leads to narrower particle distribution. The irregularity of the alloy particles could be vital for electrolyte adsorption leading to increased catalytic activity in the reduction of the electrolyte. No definite pattern can be established between the change in concentration of either component with the variance in the morphology and particle distribution depicted in the SEM images. 

**Figure 4:**
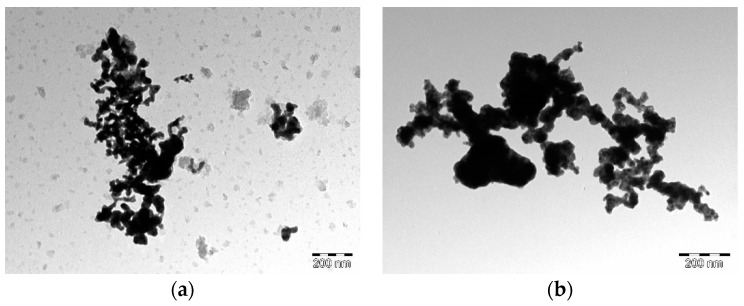

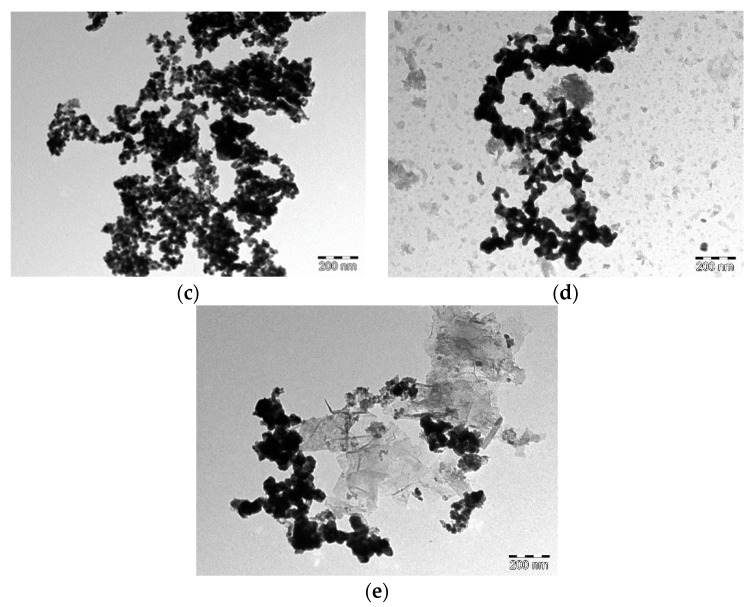
Transmission electron microscopy (TEM) images for the synthesized binary palladium PdNi_x_ alloys (**a**) PdNi_1_, (**b**) PdNi_2_, (**c**) PdNi_3_, (**d**) PdNi_4_, (**e**) PdNi-rGO.

TEM images of the palladium alloys depicted in Figure 4 show that the binary PdNi alloy nanoparticles consist of spherical, well distributed particles without any significant agglomeration at lower palladium concentrations such as in PdNi_1_ shown in Figure 4a. As the concentration of palladium initially increases significant agglomeration is observed for PdNi_2_ in Figure 4b followed by a wider particle distribution for PdNi_3_ depicted in Figure 4c. Figure 4e shows that the carbon supported PdNi-rGO alloy consisted of graphene nanosheets surrounded by a sea of PdNi alloy particles. Sizes of the binary alloy particles were determined to be 10.16, 18.09, 17.43, 9.02, and 13.55 nm for PdNi_1_, PdNi_2_, PdNi_3_, PdNi_4_ and PdNi-rGO respectively. The observed particle diameters are similar to those calculated using the Scherrer equation. The observed TEM results reveal a definite pattern between the changes in concentration to the particle morphology and distribution which could not be ascertained from the SEM results.

**Figure 5:**
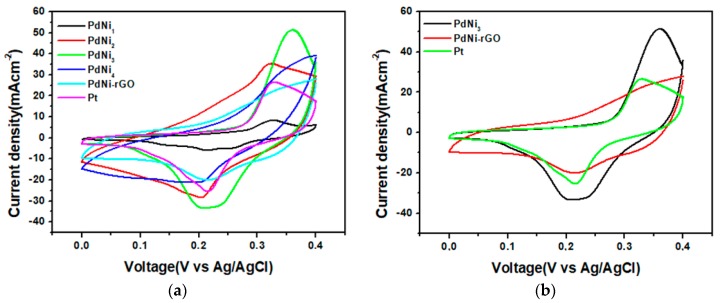

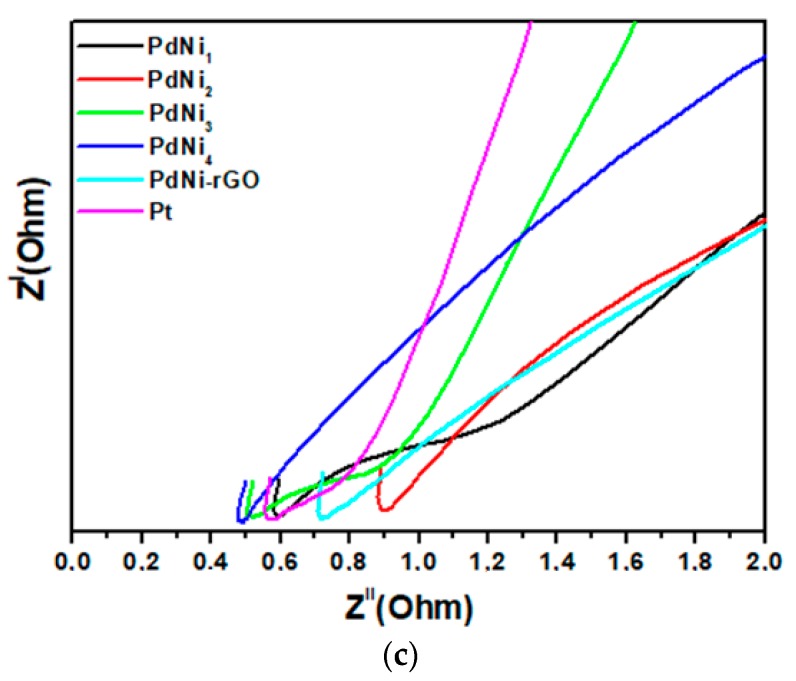
Cyclic voltammetry (CV) analysis curves for (**a**) PdNi_x_, PdNi-rGO and platinum counter electrodes, (**b**) comparison of CV curves for PdNi_x_, PdNi-rGO and platinum counter electrodes obtained at a scan rate of 50 mVs^−1^, (**c**) Nyquist plots recorded at 0V potential for PdNi_x_, PdNi-rGO and platinum.

In order to determine the electrochemical properties and the catalytic capability of the developed binary palladium samples cyclic voltammetry (CV), and electrochemical impedance spectroscopy (EIS) was conducted. Since the function of the counter electrode is to facilitate the regeneration of the electrolyte through its reduction thus the effectiveness of the developed counter electrode materials was determined through measurement of the reduction current density and peak to peak potential difference from the CV analysis as well as the charge transfer resistance from EIS. Figure 5 shows the Cyclic Voltammetry (CV) graphs for the synthesized binary palladium-nickel alloys, its carbon supported form and the platinum samples. CV analysis curves were obtained at a scan rate of 50 mV s^−1^. Two important values from CV analysis which are used to determine the catalytic effectiveness of the developed samples are the reduction current density J_p_ as well as the peak to peak potential difference ∆E_PP_. The higher the reduction current density produced during the reduction process the greater is the catalytic effectiveness of the counter electrode material. Peak to peak potential difference gives insight into the rate at which the reduction process occurred since it is inversely proportional to the rate of the reduction reaction. Consequently, potentially effective counter electrode materials must possess a low peak to peak potential difference thereby facilitating higher rates of electron transfer which diminishes the probability of electron-hole recombination’s in the dye. The reduction current density is denoted as the peak in the negative zone of the CV graph whereas peak to peak potential difference denotes the separation in potential between the two peaks in the CV curve. From Figure 5a, the increase in the reduction current density was determined to be in order PdNi_1_, < PdNi-rGO < PdNi_4_ < Pt < PdNi_2_ < PdNi_3_. The higher catalytic performance of PdNi_3_ was attributed to the existence of the optimal palladium content which facilitates the availability of sufficient active sites for effective reduction of the electrolyte. Furthermore, the wider distribution of the PdNi_3_ particles depicted in Figure 4c enables a greater area for electrolyte distribution and its easier filtration on to the counter electrode surface thereby resulting in a more effective catalytic process. As the palladium content increases upwards of the optimal level to PdNi_4_ the catalytic performance dwindles. All the CV curves for the developed samples except for PdNi-rGO consist of two peaks in the negative and positive zones corresponding to the reduction and oxidation processes of the electrolyte respectively. The absence of clear peaks and the rectangular nature of the CV curve for PdNi-rGO sample is characteristic of its non-faradaic redox capability. Since the reduction current density is a measure of the rate of electron transfer between the PdNi-rGO counter electrode surface and the electrolyte during the redox process it therefore implies that the incorporation of reduced graphene oxide on the PdNi alloy inhibits charge transfer leading to low reduction potential. This explains the low current density for the PdNi-rGO at 21 mA cm^−2^ despite the availability of the carbon support which offers higher carrier mobilities as well as a large surface area for interaction between the counter electrode surface and the electrolyte. The reduction potential for the platinum counter electrode was similarly low at 25 mA cm^−2^ as compared to 35 mA cm^−2^ and 30 mA cm^−2^ for PdNi_2_ and PdNi_3_ respectively. Peak to peak potential difference for the developed counter electrode samples increased in the order PdNi-rGO < Pt <PdNi_1_ ≈ PdNi_2_ < PdNi_4_ < PdNi_3_. This observation indicates that the rate of the reduction reaction for PdNi_3_ was the least amongst the developed counter electrode samples, which could potentially lead to greater electron-hole recombination when it is utilized as the counter electrode catalyst. Nevertheless, the lower rate of the reduction process could have potentially helped attain a high current density. A higher rate of adsorption and desorption of the electrolyte on the counter electrode surface leads to minimized and ineffective interaction with the catalytically active sites thus low reduction potentials are attained. As such optimal rate of adsorption and desorption of the electrolyte on the CE surface is required to attain the most effective catalytic conditions. Obtained results also showed that as the palladium content increases there is an initial rise in catalytic performance from the PdNi_1_ level up to the optimal level of PdNi_3_ and then subsequent decrease occurs as the palladium content increases to PdNi_4_. Figure 5c depicts the electrochemical impedance spectroscopy (EIS) analysis results for the synthesized palladium alloys as well as the platinum sample. EIS analysis gives insight into the process of charge transfer between the counter electrode and the electrolyte. Effective charge transfer is represented by a low charge transfer resistance whereas a high resistance is indicative of significant impediment to electron transportation. From Figure 5c the charge transfer resistance increases in the order PdNi_4_ < PdNi_3_ < Pt < PdNi_1_ < PdNi-rGO < PdNi_2_. The resistance for PdNi_3_ is low at 0.48 Ω which is consistent with the CV results which revealed greater electron mobility between the counter electrode and the electrolyte. The carbon supported PdNi-rGO produced a relatively high 0.74 Ω resistance characteristic of lower electron transfer capability as compared to the PdNi_3_ and the platinum counter electrode samples. Despite the excellent electrical conductivity associated with reduced graphene oxide, which is supposed to enhance carrier mobility, obtained results indicate that the incorporation of reduced graphene oxide to the palladium alloy PdNi_3_ tampers with its catalytic and conductive efficiency.

Table 2 shows the cumulative results of the electrochemical analysis clearly showing the effectiveness of PdNi_3_ as compared to its carbon supported form PdNi-rGO and the platinum counter electrode samples. As such PdNi_3_ could potentially replace the platinum counter electrode based on its higher reduction current density and lower charge transfer resistance of 35 mA cm^−2^ and 0.48 Ω as compared to 25 mA cm^−2^ and 0.56 Ω for platinum.

## 4. Conclusions

Using a simple hydrothermal procedure binary palladium alloys PdNi_x_ and its carbon supported form PdNi-rGO were successfully fabricated as alternative counter electrode catalysts to the standard platinum based counter electrode. Only palladium peaks located at 40.1°, 46.6°, 68.1°, 82.1° and 86.6° were observed in the XRD spectra for all the developed unsupported palladium samples, whilst an additional peak from PdO was observed for PdNi_4_ at 33.5°. All the developed binary palladium samples consisted of spherical particles of average diameter 10.16, 18.09, 17.43, 9.02, and 13.55 nm for PdNi_1_, PdNi_2_, PdNi_3_, PdNi_4_ and PdNi-rGO respectively. Agglomeration was observed for the PdNi_x_ samples as the palladium content initially increased to PdNi_2_ whilst wider particle distribution was prevalent in PdNi_3_ samples. Cyclic voltammetry analysis revealed that the PdNi_3_ sample performed better than all the other binary PdNi_x_ samples as well as its carbon supported form PdNi-rGO and the platinum counter electrode fabricated under similar conditions. Reduction current density, peak to peak potential difference and charge transfer resistance for PdNi_3_ were estimated at 35 mA cm^−2^, 0.15 mV, and 0.47 Ω as compared to 25 mA cm^−2^, 0.1 mV and 0.56 Ω for platinum. The lower charge transfer resistance and high reduction current density for PdNi_3_ are indicative of its superior catalytic effectiveness and higher electron transportation capability between the counter electrode and the electrolyte. Hence the developed PdNi_3_ alloy could be a potential replacement for the expensive and corrosion susceptible platinum catalyst in dye sensitized solar cell counter electrodes.

## Figures and Tables

**Table 1:** Molar quantities of reactants utilized for the fabrication of PdNi_x_ alloys.

Alloy	Molar Quantities of Reactants	
	K_2_PdCl_4_	Ni(NO_3_)_2_∙6H_2_O
PdNi_1_	0.0015	0.01
PdNi_2_	0.0024	0.0086
PdNi_3_	0.003	0.0076
PdNi_4_	0.0037	0.005
PdNi-rGO	0.003	0.0076

**Table 2:** Electrochemical parameters for the synthesized counter electrode samples.

Counter Electrode Catalyst	Reduction Current Density (J_P_)/mA cm^−2^	Peak to Peak Potential Difference (∆E_PP_)/mV	Charge Transfer Resistance (R_CT_)/Ω
PdNi_1_	7	0.11	0.58
PdNi_2_	30	0.11	0.9
PdNi_3_	35	0.15	0.47
PdNi_4_	21	0.13	0.47
PdNi-rGO	20	0.09	0.74
Platinum	25	0.1	0.56
